# Mapping Systemic Risk: Critical Degree and Failures Distribution in Financial Networks

**DOI:** 10.1371/journal.pone.0130948

**Published:** 2015-07-24

**Authors:** Matteo Smerlak, Brady Stoll, Agam Gupta, James S. Magdanz

**Affiliations:** 1 Perimeter Institute for Theoretical Physics, 31 Caroline Street North, N2L 2Y5 Waterloo ON, Canada; 2 Department of Mechanical Engineering, University of Texas at Austin, 204 E. Dean Keaton, C2200, Austin, TX 78712, United States of America; 3 Indian Institute of Management Calcutta, Diamond Harbour Road, Joka, Kolkata, West Bengal 700104, India; 4 Resilience and Adaptation Program, University of Alaska Fairbanks, PO Box 757000, Fairbanks, AK 99775-7000, United States of America; University of Bristol, UNITED KINGDOM

## Abstract

The financial crisis illustrated the need for a functional understanding of systemic risk in strongly interconnected financial structures. Dynamic processes on complex networks being intrinsically difficult to model analytically, most recent studies of this problem have relied on numerical simulations. Here we report analytical results in a network model of interbank lending based on directly relevant financial parameters, such as interest rates and leverage ratios. We obtain a closed-form formula for the “critical degree” (the number of creditors per bank below which an individual shock can propagate throughout the network), and relate failures distributions to network topologies, in particular scalefree ones. Our criterion for the onset of contagion turns out to be isomorphic to the condition for cooperation to evolve on graphs and social networks, as recently formulated in evolutionary game theory. This remarkable connection supports recent calls for a methodological rapprochement between finance and ecology.

## Introduction

In the financial sector, shock propagation mechanisms are at the core of systemic risk [1, 2], and banks play the most important role [3]. An important and potentially vulnerable arena for financial contagion is the interbank loan market, which allows banks to rapidly exchange large amounts of capital for short durations to accommodate temporary liquidity fluctuations [4–6]. In a seminal work that laid down a framework for exploring systemic risk, Eisenberg and Noe [7] developed a clearing payment algorithm for the interbank loan market and, subsequently, interbank loan networks have been of particular interest [8–10].

In recent years, random network theory [11, 12] has provided a useful framework to study contagion effects in interconnected structures [13]. Applied to the financial sector [14], network approaches have clarified the role of connectivity [15, 16], bank size [17], shock size [10] and overlapping portfolios [18] in systemic risks. Increased understanding of contagion in finance [19, 20] has led to an increased interest by regulators and central bankers [21, 22] in using network measures to evaluate systemic risk. Network centrality measures have been widely used by researchers to identify important nodes in financial networks. Battiston *et al*. [23] introduced DebtRank, a measure based on feedback centrality to identify systemically important nodes. Markose *et al*. [24] used eigenvector centrality to design a super-spreader tax to make financial systems more robust.

An essential insight of Allen and Gale [25], confirmed in [8] and deepened (with different assumptions) in [16], was that increasing network connectivity—measured by its mean degree *z*—can have opposite effects depending on the baseline value of *z*. On the one hand, when the network is sparsely connected, increasing *z* will open new channels for contagion and weaken the network. On the other hand, when *z* is sufficiently large, increasing *z* further will dilute the effect of a localized shock and strengthen the network. From this perspective, the key question is not *if*, but *when*, enhanced connectivity helps secure network robustness. Battison et. al. [26, 27], Stiglitz [28], and Roukney [29] incorporated the effects of illiquid assets and potential amplifications of failures due to human behavior in crises. These findings suggested that intermediate-degree networks would be most stable. Additionally, Battiston et. al [26] suggested in case of non-amplification that increasing degree may have an ambiguous relationship with contagion depending on the parameter values of initial robustness. However, these studies assumed a normal distribution of initial robustness. We focus on the initial failures of the system as studied by Allen and Gale—not amplifications—and study more realistic financial networks where initial distribution of robustness may not be normal.

Our first goal in this paper is to sharpen these results by introducing a model of interbank lending that allows the “critical degree” separating these two regimes to be computed as an explicit function of a small number of relevant financial parameters: (interbank and external) interest rates, liquidity requirement, leverage ratio. As we shall see, this critical degree is pivotal in deriving an analytical estimate of the number of failures induced by a single shock given these parameters. Our results complement those of [8], who used the mathematics of percolation theory [30] to determine the contagion threshold in financial networks, as well as those of [9], who brought to bear the “mean-field approximation” familiar to statistical physicists. However, the relationship between the critical degree and the economic environment is hidden in Gai and Kapadia’s work within an unspecified “probability that a bank is vulnerable”. We unpack this connection explicitly.

Our second goal is to analyze the role of degree heterogeneity in financial networks with regard to systemic risk. It has long been known [31, 32] that network topology is a key determinant of network robustness. Empirical studies of flows over the Fedwire Funds Services [33, 34] have found the network to be inhomogeneous, with a strongly connected, strongly reciprocal core and a much more sparse periphery. (Similar analyses have been conducted of interbank loan networks in Belgium [35], Austria [36], the Netherlands [37], Italy [38], and East Asia [39], with similar results.) Nonetheless, most theoretical studies of the systemic risk to date [8, 9, 25] have used homogeneous (Erdös-Rényi) networks. We show that, when banks’ degrees have a fat-tailed distribution, the number of failures induced by a single shock follows a similar distribution—a precise statement of the “robust-yet-fragile” property of financial networks emphasized by several authors [8, 10, 40].

The paper is organized as follows. We begin by describing our model of interbank lending networks, first in some generality and then under simplifying assumptions. Next we show how the number of failures induced by an individual shock can be estimated analytically by means of a mean-field-type approximation, in which Cayley trees (regular networks without loops) play an instrumental role. We then compare our results with numerical simulations of both homogenous and scalefree random networks. We close with a few remarks concerning the policy implications of our work, and point out an intriguing biological analogy.

## Results

### A network model of interbank lending

We present a model of the structure of interbank lending as a random weighted directed network, in which a node *i* ∈ {1, ⋯, *N*} represents a bank and a link *i* → *j* with weight *l*_*ij*_ a loan of amount *l*_*ij*_ made by *i* to *j*. The sum of all weights flowing out of a bank *i*, *l*_*i*_ = ∑_*j* ← *i*_ *l*_*ij*_, is therefore the total interbank exposure of bank *i*; the sum of weights flowing into *i*, *b*_*i*_ = ∑_*j* → *i*_ *l*_*ji*_, is in turn the total liability of bank *i* on the interbank market. We call “(first) neighbors” two banks which share a direct link. When a bank *j* is connected to another bank *i* through a path of length larger than one, we say that *j* is a “higher order neighbor” of *i*.

In addition to its interbank liabilities *b*_*i*_, we assume that each bank *i* has external, more senior liabilities *s*_*i*_ (e.g. deposits). We assume that all of these (interbank and senior) liabilities will be available for reinvestment in external investment opportunities at a later time period. On the asset side, we further introduce liquid assets *λ*_*i*_ (e.g. bonds) as well as illiquid assets *ι*_*i*_ (e.g. buildings). The total assets *A*_*i*_ and total liabilities *L*_*i*_ of bank *i* can therefore be written as *A*_*i*_ = *l*_*i*_+*λ*_*i*_+*ι*_*i*_ and *L*_*i*_ = *b*_*i*_+*s*_*i*_; the difference *K*_*i*_ = *A*_*i*_−*L*_*i*_ is the net worth of bank *i*, see Table 1.

**Table 1:** Initial balance sheet of bank *i*.

assets *A*_*i*_	liabilities *L*_*i*_
liquid assets *λ*_*i*_	senior liabilities *s*_*i*_
illiquid assets *ι*_*i*_	interbank borrowings *b*_*i*_
interbank loans *l*_*i*_	net worth *K*_*i*_

Basel III [41] introduced leverage and liquidity requirements for banks. We define for each bank *i* the *leverage ratio* Λ_*i*_ = *K*_*i*_/*A*_*i*_ (ratio of networth to total assets) and the *liquidity ratio* *f*_*i*_ = *λ*_*i*_/*A*_*i*_ (ratio of liquid assets to total assets). By definition, lowering the ratios Λ_*i*_ and *f*_*i*_ increases the exposure of bank *i* on the interbank market; we shall see that they have a strong impact on the systemic risk.

We now introduce a discrete-time investment dynamic, through which a bank can either increase or decrease its net worth *K*_*i*_. We assume that the investment period is shorter than the time needed to liquidate illiquid assets. The period begins with the balance sheet introduced in Table 1. In the first step, a bank uses its initial total liabilities *L*_*i*_ to invest in some external opportunity, at some interest rate *R*_*i*_. (Successful investments correspond to *R*_*i*_ > 1, hazardous ones correspond to *R*_*i*_ < 1; in the worst case scenario, the investment is lost in full, viz. *R*_*i*_ = 0.) We denote *ρ*_*i*_ = (*R*_*i*_ − 1)*L*_*i*_ the profit made in this transaction. (If a bank only borrows and does not lend, *b*_*i*_ > 0 and *l*_*i*_ = 0, we take *ρ*_*i*_ = (*R*_*i*_ − 1)*b*_*i*_; equivalently, the profit is defined by *ρ*_*i*_ = (*R*_*i*_ − 1)max{*L*_*i*_, *b*_*i*_}.) In the second step, a bank uses this profit and its liquid assets *λ*_*i*_ to repay its interbank liabilities *l*_*i*_ with an interest *r* > 1 while ensuring the seniority of *s*_*i*_. When a bank *i* can just repay its interbank borrowings, we say that *i* is *critical*. (See Methods for details.)

From a mathematical perspective, finding the interbank repayments *x*_*i*_ (a.k.a. the clearing vector) amounts to solving the system of *N* coupled, non-linear equations

$$\begin{array}{c} x_{i}={\left[\min\{\rho_{i}+\lambda_{i}-s_{i}+\sum_{j\leftarrow i}\left(l_{ij}/b_{i}\right)x_{j},rb_{i}\}\right]}^{+}, \end{array}$$ (1)

where [⋅]^+^ = max{ ⋅, 0} and the sum ranges over *i*’s debtors; the repayment *x*_*ij*_ of bank *i* to bank *j* is then given by *x*_*ij*_ = *l*_*ij*_ *x*_*i*_/*b*_*i*_, as first proposed by Eisenberg and Noe [7]. After all repayments are made, bank *i* has an updated net worth

$$\begin{array}{c} K_{i}^{\prime }=\rho_{i}+\lambda_{i}+\iota_{i}-s_{i}+\sum_{j\neq i}\left(x_{ji}-x_{ij}\right). \end{array}$$ (2)

We call *safe* the banks *i* such that $K_{i}^{\prime }>0$, and *failed* the ones such that $K_{i}^{\prime }\leq 0$.

While the set of Eq (1) can be studied numerically for various network topologies and different values of the financial parameters *R*_*i*_, *f*_*i*_, Λ_*i*_ and *r*, our goal in this paper is to obtain explicit, analytical results about the robustness of financial networks with respect to shocks. To make progress, we make the following—dramatic but empowering—assumptions: (*i*) all loans are reciprocated, so that the network is actually *undirected* and loans are made in the same amount in both directions, (*ii*) all interbank loans *l*_*ij*_ have unit value, so that *l*_*i*_ = *b*_*i*_ = *k*_*i*_, where *k*_*i*_ is the degree of node *i*, (*iii*) all banks have equal leverage and liquidity ratios (Λ, *f*), so that the latter can be thought of as model parameters rather than individual variables (*iv*) illiquid assets are negligible (*ι*_*i*_ = 0), and (*v*) external interest rates *R*_*i*_ take the same value *R* > 1 for all banks across the network except one (bank *i* = *i*_0_, call it the “shocked bank”), for which *R*_*i*_0__ = 0.

The reciprocity of the “core” of real financial networks is very high [33], making our first assumption less unrealistic than may seem at first sight. Garlaschelli [42] found that real networks are typically highly reciprocal or highly areciprocal, and Musmeci [43] determined that exposure is related to topology. Networks with non-reciprocal links provide an example of an interesting extension of this work. The assumption of unitary loans is made in order to provide a simplistic yet analytical solution. This allows for general lessons to be learned from our analysis, rather than more realistic but less widely applicable solutions. Simplistic assumptions to enhance analytical tractability have often been used in finance literature. For example, Freixas et al [44], Aghion et al. [19], Diamond and Dybvig [45] and Postlewaite and Vives [46] have all made simplifying assumption about unitary deposits to study individual bank insolvency. We additionally analyzed perturbations in this assumption, see Supporting Information for details.

Our third assumption represents a worst case scenario in which all banks within a financial network are operating at the limit of regulatory caps, such as the Basel norms. The fourth assumption is due to the model set up, whereby the two periods we analyze are much shorter than the time in which illiquid assets could be liquidated. The assumption of uniform external interest rates is made based on an assumption that the banks are operating in a similar environment.

Our model then creates a single shock to the system, in the form of one bank losing its external investement. Due to interbank lending, all other banks in the network can in principle feel the effects of this shock, either directly (first neighbors, direct creditors) or indirectly (higher order neighbors, indirect creditors). Within this setting, our objective is to estimate the understand the onset of contagion, in particular through the *number of induced failures* *F* (the number of banks *i* ≠ *i*_0_ such that $K_{i}^{\prime }\leq 0$), as a function of the financial parameters (*R*, *r*, Λ, *f*) and of the network topology.

### Failures on Cayley trees

We begin our investigation of the model by considering the simplest network topology, namely a network with uniform degree *k* and no loops (a “Cayley tree”). On such simple networks, the repayment problem (1) can be solved exactly as follows (see Methods and Supporting Information for details).

When *k* is sufficiently large, each neighbor of the shocked bank *i*_0_ inherits only a small fraction of *i*_0_’s losses—and none fails. For networks with incrementally decreasing degree *k*, however, the effect of these losses on the net worth of each creditor of *i*_0_ gradually increases, until at some point shocked bank *i*_0_’s weakest neighbor also fails. If degree *k* further decreases, the second (then third, etc.) order neighbors of *i*_0_ also approach criticality, and start failing as well.

This sequence of transitions, involving higher and higher order neighbors of the shocked bank, defines an ordered sequence of “critical degrees” $k_{\left(1\right)}^{*}>k_{\left(2\right)}^{*}>...$ such that

$$\begin{array}{c} F=\sum_{p=1}^{q}N_{\left(p\right)}\left(k\right)\;\text{for}\;k_{\left(q+1\right)}^{*}<k\leq k_{\left(q\right)}^{*} \end{array}$$ (3)

where *N*_(*p*)_(*k*) = *k*(*k*−1)^*p*−1^ is the number of nodes at distance *p* from *i*_0_. The values of these critical degrees provide a measure of the robustness of the network with respect to a shock: the higher the critical degrees, the more fragile the financial structure.

The expression for each $k_{\left(q\right)}^{*}$ as a function of the financial parameters (*R*, *r*, *f*, Λ) can be obtained by solving the repayment Eq (1) under these conditions that (*i*) all banks at distance *d* ≥ *q* from the shocked bank are safe, but (*ii*) all *q*-th neighbors of *i*_0_ are critical. This gives in particular

$$\begin{array}{ccc} k_{\left(1\right)}^{*} & = & \frac{r\left(1-f\right)-{\left[r(1-f)+2\Lambda -1\right]}^{+}}{(R-1)(1-\Lambda )+\Lambda }. \end{array}$$ (4)

**Fig 1:**
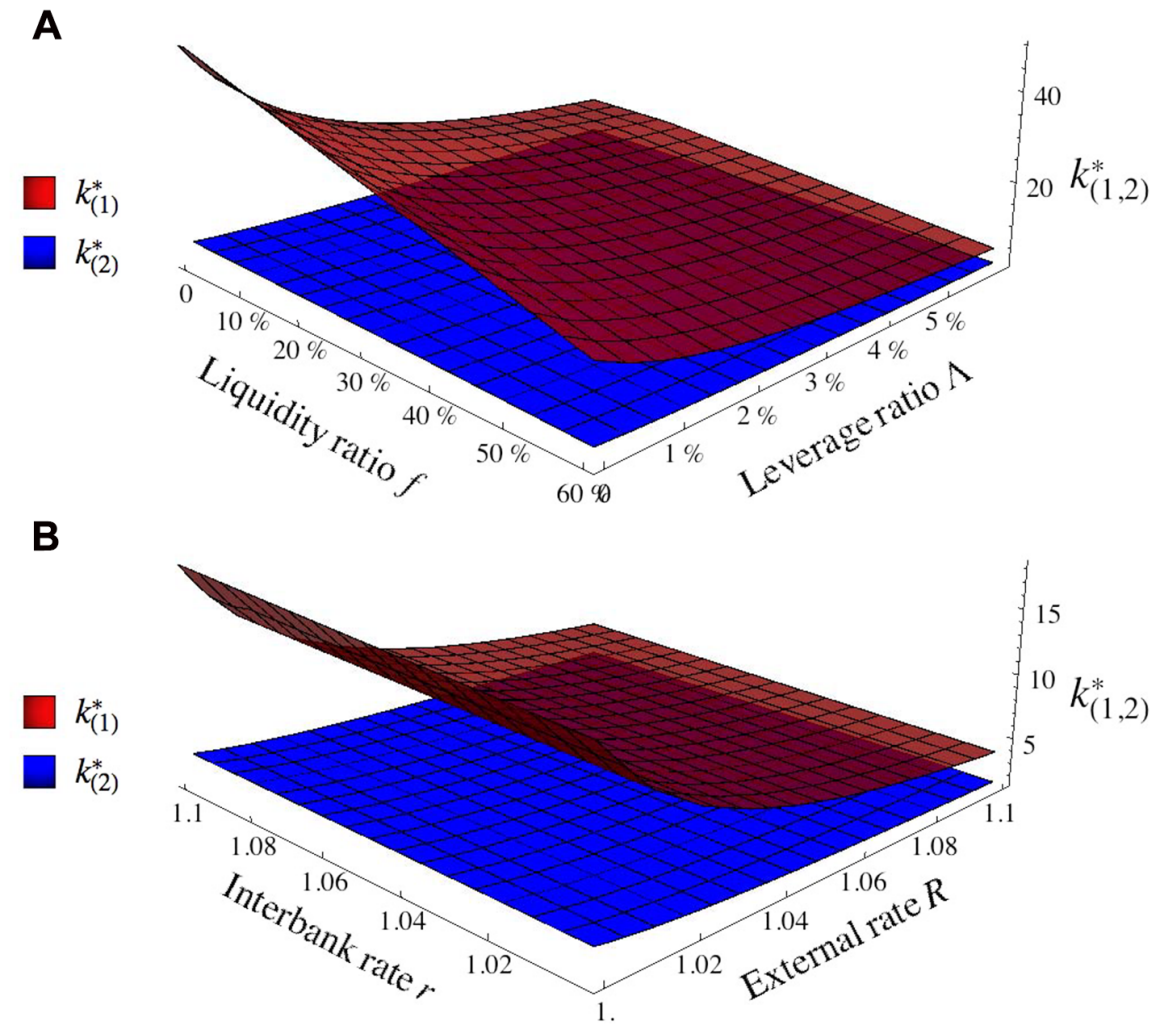
The first two critical degrees $k_{\left(1\right)}^{*}$ and $k_{\left(2\right)}^{*}$ as functions of the liquidity ratio *f* and of the leverage ratio Λ for *R* = 1.05, *r* = 1.01 (Fig 1a) and as functions of the external rate *R* and the interbank rate *r* for *f* = 50% and Λ = 3% (Fig 1b).

The critical degrees $k_{\left(1\right)}^{*}$ and $k_{\left(2\right)}^{*}$ (given explicitly in Supporting Information) are plotted as functions of the financial ratios (*f*, Λ) for *R* = 1.02 and *r* = 1.01 and as functions of the interest rates (*R*, *r*) for *f* = 50% and Λ = 3% in Fig 1. As is apparent from these plots, $k_{\left(1,2\right)}^{*}$ are stricly decreasing functions of *f* and Λ: lower liquidity and leverage ratios both enhance the systemic risk—an intuitive conclusion, which is here proved rigorously. Furthermore, we see that, unlike the first critical degree $k_{\left(1\right)}^{*}$, the second critical degree never becomes appreciably large, $k_{\left(2\right)}^{*}≲5$, so that failures in effect hardly extend beyond the first neighbors of the shocked bank. Finally, we note that, in the limit where Λ, *f* → 0 (a regime in which the economy is dominated by interbank transactions), the first critical degree $k_{\left(1\right)}^{*}$ reaches the value 1/(*R* − 1); we will come back to this observation in the concluding section.

### Failures on general networks

Real-world financial networks being anything but regular, the usefulness of the exact solution above would seem to be extremely limited. It turns out to be the opposite. In the regime where failures are unlikely to extend beyond the first neighbors of the shocked bank—which is the case for most realistic values of the financial parameters, as illustrated by the low values of $k_{\left(2\right)}^{*}$ in Fig 1—knowing the first critical degree (hereafter denoted simply k*) yields a reliable estimate of the distribution of number of failures *P*(*F*) (hereafter “failures distributions”) on random networks, including scalefree ones. We focus our analysis on this regime.

We will assume that, on a general random network, a first neighbor *i* of the shocked bank will indeed fail if and only if its own degree *k*_*i*_ is smaller than the critical degree k* given by Eq (4). This is akin to the “mean-field” approximation familiar from statistical mechanics: it replaces the actual, inhomogeneous, environment of *i* in the network by a homogeneous environment in which all banks have the same degrees as *i*, here the Cayley tree with degree *k*_*i*_. While this approximation clearly cannot capture all the dynamics of a single network, it does provide a tractable starting point to study the statistics of failure contagion in a given ensemble of random networks. Within this approximation, we obtain the following results (see Methods and Supporting Information for details).

First, we have an explicit lower bound on the expected number of failures

$$\begin{array}{c} \left\langle F\right\rangle \geq \sum_{k\geq 1}kp\left(k\right)q\left(k\right), \end{array}$$ (5)

where $q(k)=\sum_{l=1}^{k^{*}}p(l|k)$ is the probability that a neighbor of the shocked bank *i*_0_ has a subcritical degree. Here *p*(*k*) is the degree distribution (probability that a node has degree *k*) and *p*(*l*|*k*) is the conditional degree distribution (probability that a node attached to a node with degree *k* has degree *l*). The positive remainder ⟨*F*⟩−∑_*k* ≥ 1_ *kp*(*k*)*q*(*k*) corresponds to the contribution of higher order neighbors, which is neglected here. Note that formula (5) implies that disassortative financial networks (for which the probability *q*(*k*) that a neighbor of the shocked bank has subcritical degree increases with the number of neighbors *k*) tend to be more vulnerable to contagion than assortative or uncorrelated ones [32]. (In the latter case, one checks that Eq (5) reduces to $\langle F\rangle =\sum_{l=1}^{k^{*}}lp(l)=qz$, where *q*(*k*) = *q* is independent of *k*.)

Second, we show that, whether the network is Poisson-distributed (*p*(*k*) ∼ *z* ^*k*^/*k*!) or power-law distributed (*p*(*k*) ∼ *k* ^−*γ*^), the failures distributions *P*(*F*) has the same asymptotic behavior as the degree distribution itself. This can be understood as follows. This is because, for the number of failures *F* to be large in the regime where only first neighbors of the shocked bank can fail, one needs: (*i*) the shocked bank *i*_0_ to have large degree *k*, and (*ii*) many neighbors of *i*_0_ to have subcritical degree *l* ≤ k*. The correlation of *k* and *l* being a decreasing function of |*k* − *l*|, these two conditions become independent in the limit *k* ≫ k* ≥ *l*, in which case the failures distribution *P*(*F*) simply reflects the degree distribution *p*(*k*). In the scalefree case, this means in particular that *P*(*F*) has a power-law falloff with exponent *γ*, hence is *fat tailed*. This result can be interpreted as expressing the “robust-yet-fragile” property of scalefree networks noted earlier: even when the expected number of failures ⟨*F*⟩ is low, the risk remains that a single shock can take down a significant fraction of the network.

### Numerical tests

**Fig 2:**
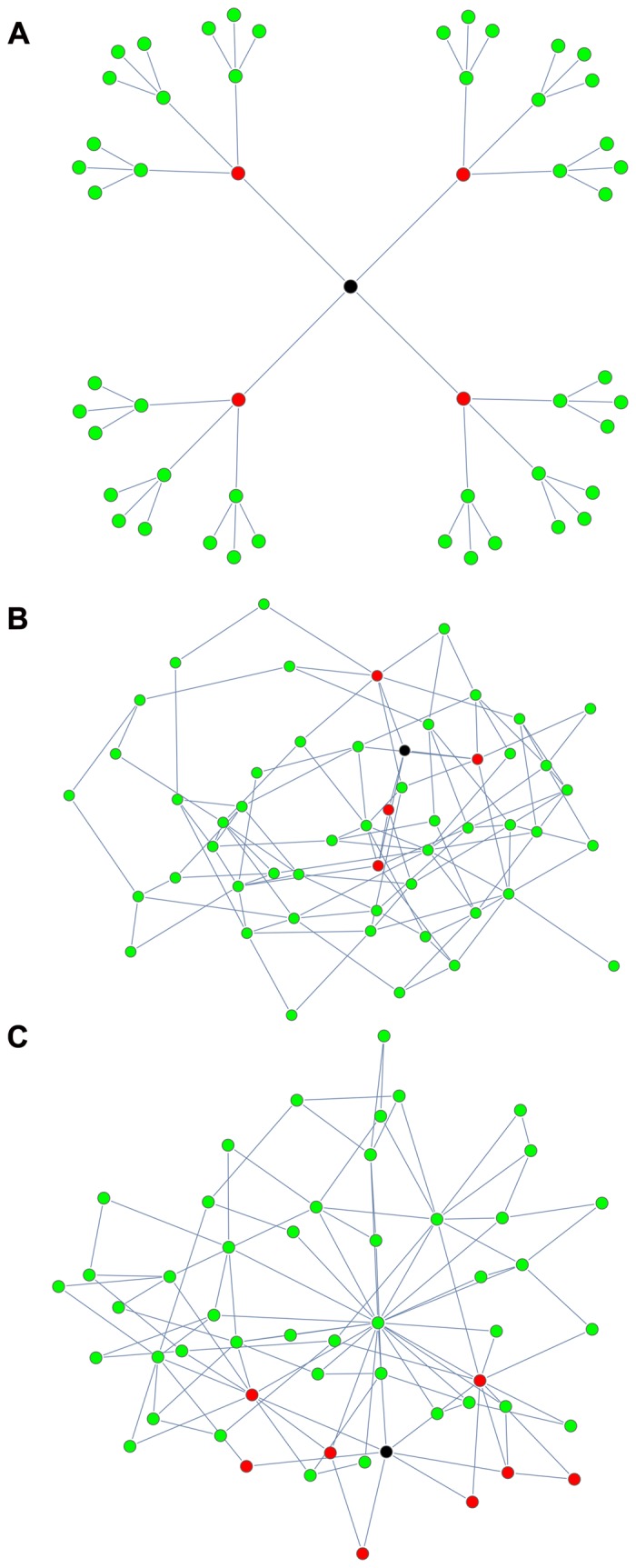
Failures in sample networks with *N* = 53 and *z* = 4: Cayley tree (Fig 2a), ER network (Fig 2b), BA network (Fig 2c). The black node indicates the shocked bank, the red nodes the failed banks, the green nodes the safe banks. Here *R* = 1.02, *r* = 1.01, *f* = 50% and Λ = 3%.

To test the validity of these findings, we analyzed two additional types of random networks for which the conditional probability distribution *p*(*l*|*k*) is known explicitly as a function of the mean degree *z* (at least in the large *N* limit): the classical Erdös-Rényi (ER) model [47], with Poisson degree distribution, and the Barabási-Albert (BA) model [48], with scalefree degree distribution *P*(*k*) ∼ *k* ^−3^, see Fig 2.

For both network types, we generated 10^4^ random networks for each value of the mean degree *z*. We solved Eq (1) numerically for each of these networks and, using Eq (2), we computed the number of induced failures *F* over the whole network. From this we determined the mean number of failures over all of the randomly generated networks and the empirical failures distribution at each *z*. We compared these distributions with our mean-field bounds. Finally, we checked that using directed networks (both random and scalefree) does not yield significantly different results, thereby confirming the validity of assumption (*i*) as a useful first approximation.

**Fig 3:**
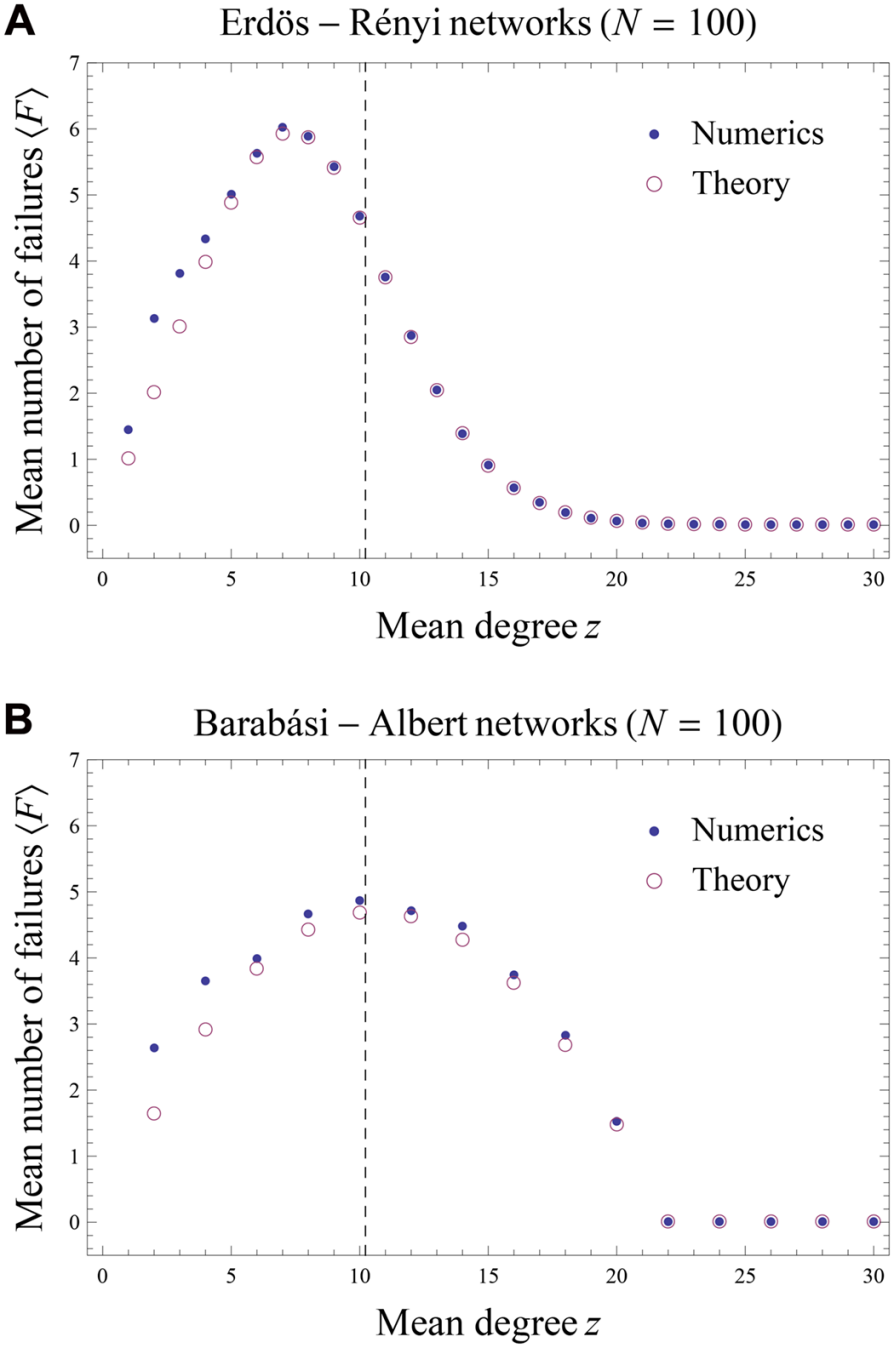
Mean number of failures as a function of mean degree *z*, as estimated analytically (circles) and as obtained numerically (dots), for ER networks (Fig 3a) and BA networks (Fig 3b). The dashed line indicates the value of the critical degree k*. The discrepancy between the empirical and theoretical values at low *z* is due to the contribution of second neighbors, neglected in our approximation.

Fig 3 shows the mean number of failures ⟨*F*⟩ as a function of the mean degree *z* for homogeneous ER and scalefree BA networks, in the parameter regime *R* = 1.02, *r* = 1.01, *f* = 50% and Λ = 3% (for which k* ≃ 10.2); see also Figure B in S1 Text. Irrespective of the network topology, we find that the empirical value of ⟨*F*⟩ matches very closely with our estimate Eq (5) provided that the mean degree is not too small. This discrepancy at low *z* has a straightforward explanation: while we neglected their contribution in our mean-field approximation, we saw with Cayley trees that the likelihood of second and higher order neighbors failing is a decreasing function of *z*.

**Fig 4:**
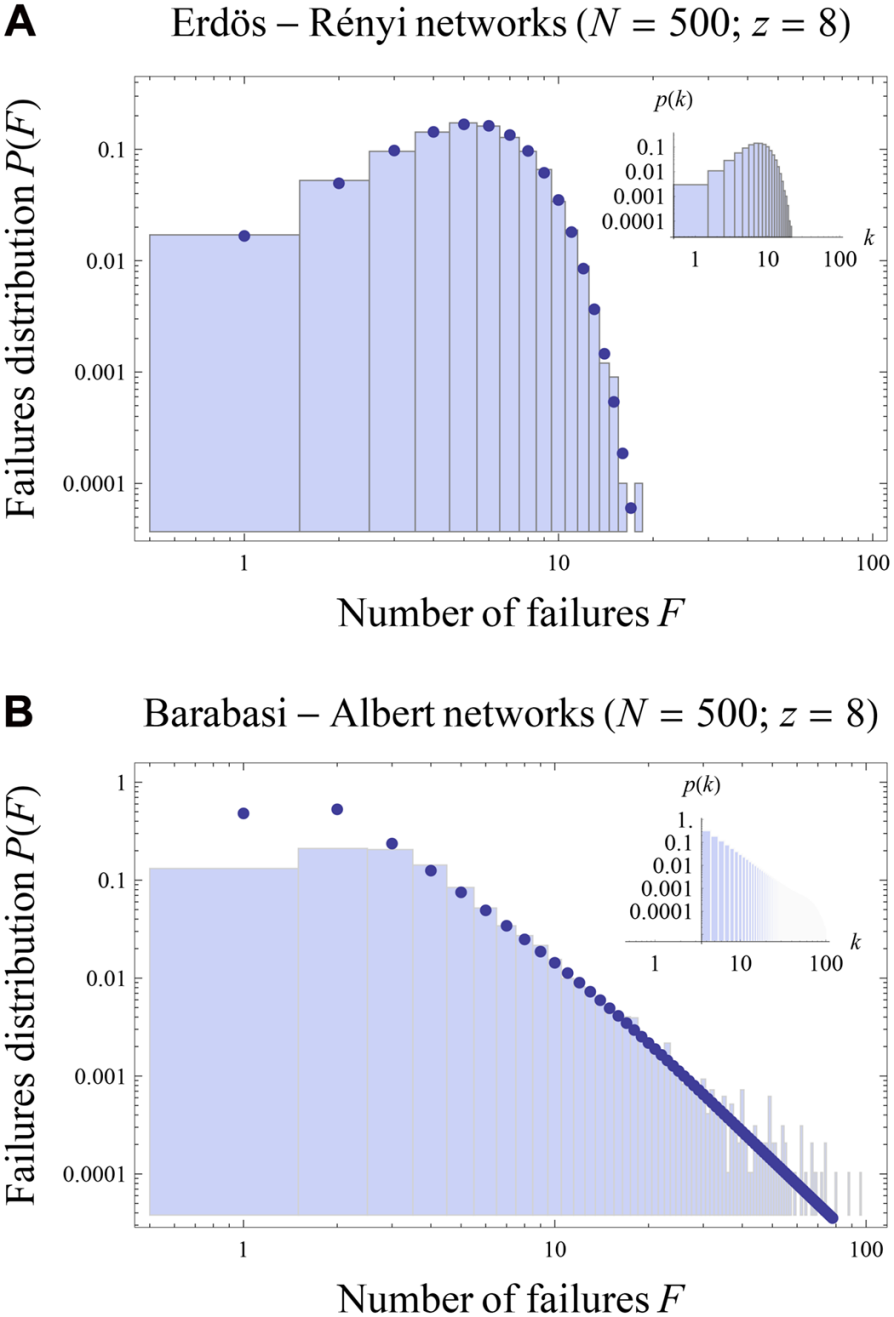
Statistics of failures in ER networks (Fig 4a) and BA networks (Fig 4b), for *R* = 1.02, *r* = 1.01, *f* = 50% and Λ = 3%. Observe the “robust-yet-fragile” nature of scalefree networks: while the maximum expected number of failures is lower than for ER networks, the probability of catastrophic failures is much higher.

Fig 4, in turn, plots the empirical distribution of failures *P*(*F*) and our analytical estimate thereof (given in Methods) for ER and BA random networks with *z* = 8, for the same values of the financial parameters. Here too, we find that the agreement between the numerical results and the prediction of our mean-field approximation is very good; Fig 4b confirms in particular that *P*(*F*) is fat-tailed when *p*(*k*) is (for BA networks).

The close agreement for these values of the financial parameters (and any other values such that k* ≲ 15, see Supporting Information) is remarkable if one contrasts the complexity of the original problem (1) with the extreme simplicity of our mean-field approximation. To us, this conclusion is the main import of our study: once the expression for the critical degree k* as a function of the financial parameters has been obtained, Eq (4), analytical results on the systemic risk are not only possible, but also intuitive and straightforward.

## Discussion

We have considered the effect of financial variables such as interest rates, leverage ratio and financial exposure on the robustness of interbank systems vis-à-vis individual shocks. Focusing first on regular networks, we obtained an explicit formula for the critical degree, below which failures begin to propagate through the network. From this, we then showed how to derive a simple but reliable lower bound on the expected number of failures and failures distribution in random (and possibly strongly heterogeneous) networks. Besides an in-depth study of cascades beyond first neighbors of the shocked bank, interesting extensions of our work could include a non-linear relation between interbank exposure and network degree, overlapping portfolios, multiple or probabilistic shocks, multiple-period dynamics, and amplifications of failures. Additionally, our assumptions, particularly reciprocal loans, could be relaxed to more realistically represent real-world networks.

The highly stylized character of our model notwithstanding, our results shed new light on important aspects of systemic risks, such as the association between contagion and interest rate policy [44]. Using plausible values for interest rates, liquidity requirement and leverage, we found critical degrees k* of the order of 5 to 10. An empirical analysis of the FedFunds market [33] found a mean degree *z* ≃ 15, but almost half of the banks had out-degrees less than 4, thus vulnerable to contagion. As mentioned previously, $k_{\left(2\right)}^{*}$ is nearly always < 5, and therefore can significantly impact contagion. In the 2008 financial crisis, mean degrees in the interbank network declined, increasing systemic risk [49]. While regulators do not directly control the topology of financial networks, it is useful to understand how tools already in place—interest rates, leverage and liquidity requirements—can affect the critical degree.

We observed that our formula (4) for the critical degree k* reduces to 1/(*R* − 1) in the high leverage, low liquidity limit. This limiting value can be expressed as a “cost-benefit” rule of thumb, as follows. If bank *i* lends *l* to bank *j* and *j* does not repay *i*, *i* will have lost *l*; if on the other hand *j* makes a successful investment with the money borrowed from *i* and repays it in full, *j* will have made a profit profit (*R* − 1)*l*. The critical degree k* is then just the ratio of the potential loss *l* to the potential profit (*R* − 1)*l* of each transaction. Given the great difficulty of the problem of assessing the robustness of actual financial networks, this simple rule of thumb could prove a handy “order-zero” approximation.

What is more, this interpretation establishes a direct link with a seemingly unrelated problem: the condition for the evolution of cooperation, famously investigated by Hamilton [50]. Ref. [51] recently extended his insights to graphs and social networks, showing that “natural selection favours cooperation, if the benefit of the altruistic act, *b*, divided by the cost, *c*, exceeds the average number of neighbours, *k*, which means *b*/*c* > *k*”. This simple rule is precisely the same as the one we found for shock propagation in high-leverage, low liquidity interbank networks: the critical degree is given by the ratio of the activities promoting systemic propagation (benefit of cooperation and interbank lending respectively) to the activities inhibiting systemic propagation (cost of cooperation and external profits respectively). This unexpected connection supports the convergence of ecology and finance advocated by Haldane and May after the 2008 crisis [52], and points to a unified perspective on resource sharing in networks.

## Methods

### Repayment equations

Denoting *x*_*ij*_ the amount repaid by bank *i* to bank *j* in the second step, we assume the following repayment rules.

- *Full repayment*: if *ρ*_*i*_+*λ*_*i*_−*s*_*i*_+∑_*j* ≠ *i*_ *x*_*ji*_ ≥ *rb*_*i*_, bank *i* repays its junior debt *rb*_*i*_ in full, hence for each *j* ≠ *i*
  $$\begin{array}{c} x_{ij}=rl_{ji}, \end{array}$$
- *Partial default*: if 0 < *ρ*_*i*_+*λ*_*i*_−*s*_*i*_+∑_*j* ≠ *i*_ *x*_*ji*_ < *rb*_*i*_, bank *i* repays a fraction of its junior liabilities on a *pro rata* basis, hence for each *j* ≠ *i*
  $$\begin{array}{c} x_{ij}=\frac{l_{ji}}{b_{i}}(\rho_{i}+\lambda_{i}-s_{i}+\sum_{j\neq i}x_{ji}) \end{array}$$
- *Complete default*: if *ρ*_*i*_+*λ*_*i*_−*s*_*i*_+∑_*j* ≠ *i*_ *x*_*ji*_ ≤ 0, bank *i* repays nothing, hence *x*_*ij*_ = 0 for each *j* ≠ *i*.

We call *critical* a bank *i* such that

$$\begin{array}{c} \rho_{i}+\lambda_{i}-s_{i}+\sum_{j}\left(l_{ij}/b_{i}\right)x_{j}=rb_{i}. \end{array}$$ (6)

### Networks

In this paper we considered three classes of networks: Cayley trees, ER networks and BA networks. They are defined as follows.

- Cayley trees are graphs without loops in which each node is connected to a fixed number of neighbors *k*. Given an (arbitrarily chosen) “root” node *i*_0_, the number of nodes at distance *d* from *i*_0_ is *k*(*k* − 1)^*d*−1^.
- ER networks are the simplest random networks: given *N* nodes, each possible edge is included in the network with probability *ϕ*, independently from every other edge. When *N* ≫ 1, this results in a random network with Poisson degree distribution
  $$\begin{array}{c} p\left(k\right)=e^{-z}\frac{z^{k}}{k!}, \end{array}$$ (7)
  where *z* = *ϕ*(*N* − 1) is the mean degree. The absence of correlations in such networks entails that the conditional degree distribution—the probability that a node connected to a node with degree *k* has degree *l*—is just *p*(*l*|*k*) = *lp*(*l*)/*z*.
- BA networks are obtained by means of a stochastic growth process. Starting from a complete graph over (say) *m* initial nodes, each new node is added to *m* existing nodes with a probability that is proportional to the number of links that the existing nodes already have. In the large time limit, this process defines a correlated random network with (conditional) degree distribution [53]
  $$\begin{array}{c} p\left(k\right)=\frac{2m(m+1)}{k(k+1)(k+2)}, \end{array}$$ (8)
  $$\begin{array}{c} p\left(l|k\right)=\frac{m}{kl}(\frac{k+2}{l+1}-(\begin{array}{c} 2m+2\\ m+1 \end{array})\frac{\left(\begin{array}{c} k+l-z\\ l-m \end{array}\right)}{\left(\begin{array}{c} k+l+2\\ l \end{array}\right)}), \end{array}$$ (9)
  where *k* ≥ *m*; the mean degree is given by *z* = 2*m*.

### Distribution of first-neighbors failures

Here we estimate the probability *P*_1_(*F*_1_) that *F*_1_ first neighbors of the shocked bank fail. Clearly, the probability that *F* banks fail throughout the network (in addition to the shocked bank *i*_0_ itself) is larger than the probability that *F* first neighbors of *i*_0_ fail, i.e. *P*(*F*) ≥ *P*_1_(*F*). In the main text we made the approximation *P*(*F*) ≃ *P*_1_(*F*) and confirmed its validity numerically.

Consider a random network with degree distribution *p*(*k*) and conditional degree distribution *p*(*k*|*l*). Suppose that the shocked bank *i*_0_ has degree *k*, and let $q(k)=\sum_{l=1}^{k^{*}}p(l|k)$ be the probability that a neighbor of the shocked bank *i*_0_ has a subcritical degree. According to our “mean-field” assumption, the probability that *F*_1_ neighbors of *i*_0_ fail is given by the probability $q{\left(k\right)}^{F_{1}}$ that *F*_1_ neighbors have subcritical degree, times the probability ${\left[1-q(k)\right]}^{k-F_{1}}$ that *k* − *F*_1_ first neighbors have supercritical degree, times the number of choices of *F*_1_ failing neighbors among *k*. Weighing this by the probability *p*(*k*) that *i*_0_ has *k* neighbors, we arrive at

$$\begin{array}{c} P_{1}\left(F_{1}\right)=\sum_{k\geq F_{1}}p\left(k\right)(\begin{array}{c} k\\ F_{1} \end{array})q{\left(k\right)}^{F_{1}}{\left[1-q(k)\right]}^{k-F_{1}}. \end{array}$$ (10)

The expected number of first-neighbor failures is then obtained by evaluating ⟨*F*_1_⟩ = ∑_*F*_1_ ≥ 1_ *F*_1_ *P*(*F*_1_) (see Supporting Information). Note that expression (10) is strongly reminiscent of the classical theory of percolation on complex networks, where one shows [54] that the degree distributions *p*′(*k*′) after the removal of a fraction *q* of the nodes is given in terms of the old degree distribution *p*(*k*) by $p^{\prime }(k^{\prime })=\sum_{k\geq k^{\prime }}p(k)\left(\begin{array}{c} k\\ k^{\prime } \end{array}\right)q^{k}{\left(1-q\right)}^{k^{\prime }-k}$. This is no surprise: the whole point of our mean-field approximation is to reduce a dynamical problem (computation of repayments) to a topological one (failure depends on degree only).

### Large degree asymptotics

Let us now consider the limit of Eq (10) when *F*_1_ ≫ 1 (hence for shocked banks with degree *k* ≫ 1), assuming that *q*(*k*) becomes independent of *k* in this limit. (This amounts to saying that correlations between the degrees *k* and *l* of adjacent nodes become immaterial when |*k* − *l*| ≫ 1; this holds for both ER and BA networks.) Let us consider Poisson-distributed and power-law distributed networks separately (see Supporting Information for details).

- *Poisson networks*. Given the Poisson degree distribution *p*(*k*) = *e* ^−*z*^ *z* ^*k*^/*k*!, resumming Eq (10) is straighforward and gives
  $$\begin{array}{c} P_{1}\left(F_{1}\right)=e^{-zq}\frac{{\left(zq\right)}^{F_{1}}}{F_{1}!}. \end{array}$$ (11)
  Thus, in Poisson distribued networks, the failures distribution is Poissonian with mean *zq*.
- *Scalefree networks*. For scalefree networks we observe that, when *γ* is an integer and for sufficiently large *k*, the Pochhammer symbol (*k*)_*γ*_ = *k*(*k*+1)…(*k*+*γ* − 1) can be substituted to *k* ^*γ*^ in the degree distribution *p*(*k*) ∼ 1/*k* ^*γ*^. This allows to perform the sum (10) explicitly, yielding
  $$\begin{array}{c} P_{1}\left(F_{1}\right)∼\frac{q^{F_{1}}\;{}_{2}{𝓕}_{1}\left(F_{1},F_{1}+1;F_{1}+\gamma ;1-q\right)}{{\left(F_{1}\right)}_{\gamma }}∼\frac{q^{\gamma -1}}{F_{1}^{\gamma }}, \end{array}$$ (12)
  where _2_𝓕_1_ is the Gauss hypergeometric function, whose asymptotics for large parameters is given in [55]. Analytical continuation in *γ* then shows that *P*_1_(*F*_1_) is scalefree with exponent *γ* also for non-integer *γ*.

In both cases, the tail of *P*_1_(*F*_1_) has the same nature (Poisson or power-law) as the degree distribution itself.

## Supporting Information

S1 Text Computation of critical degrees, mathematical proofs and further numerical results, including the effect of loan asymmetry (Figure A) and varying interest rates and leverage ratios (Figure B). (PDF)
