# Supplementary material for: Mapping Systemic Risk: Critical Degree and Failures Distribution in Financial Networks
Source: PLoS One. 2015 Jul 24;10(7):e0130948. doi: 10.1371/journal.pone.0130948 (PMC4514837; doi:10.1371/journal.pone.0130948)
Supplement: S1 Text — (PDF) [file pone.0130948.s001.pdf]

Mapping systemic risk: critical degree and failures distribution in financial networks

Supplementary Information

Matteo Smerlak, Brady Stoll, Agam Gupta and James S. Magdanz

Perturbations in symmetrical loans

For the above analysis we have assumed symmetrical loan weights throughout the network. This is a strong assumption, so to determine the accuracy of this assumption we have analyzed asymmetrical loan weight and the impact of asymmetrical loans on the failures distribution.

We specifically analyzed Cayley trees with degree $k \in \{2, \cdots 6\}$. For each link (i, j), the weight of a loan l_{ij} was drawn uniformly in $[1-\epsilon, 1+\epsilon]$. We analyzed 4 values of ϵ, from 0.1 to 0.4, in addition to $\epsilon = 0$. This was done for 10^2 networks for each degree k and perturbation ϵ, and the resulting number of failures was measured. Figure A shows the results for $R = 1.02$, $r = 1.01$, $f = 50\%$ and for two values of Λ, namely $\Lambda = 1\%$ (left) and $\Lambda = 3\%$ (right), for which $(k*_{(1)}, k*_{(2)}) = (16, 3)$ and $(k*_{(1)}, k*_{(2)}) = (10, 2)$ respectively.

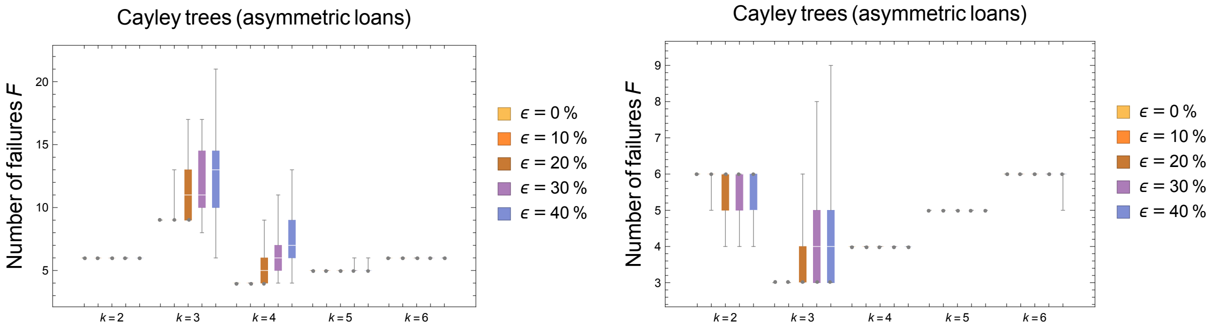

Figure A: Number of failures on a Cayley tree using asymmetric loans. Loan weight was perturbed by value ϵ for each degree k shown. Here $R = 1.02$, $r = 1.01$, $f = 50\%$ and $\Lambda = 1\%$ (left) and $\Lambda = 3\%$ (right).

The figures show a small variance in number of failures for many values of k across all values of ϵ, and at other particular values of k show a high variance. These values of k are close to the value of $k*_{(2)}$, which leads to significant exposure risk for second degree neighbors, particularly as ϵ increases. As the asymmetry of a network increases, the vulnerability of second neighbors for $k \simeq k*_{(2)}$ is increased. This notwithstanding, our assumption of symmetrical loans is a reasonable first approximation that can provide valuable insight on the overall behavior of failures at mean degrees larger than $k*_{(2)}$.

Computation of the critical degrees $k^*_{(1)}$ and $k^*_{(2)}$

On a regular graph with degree k, the liquid assets λ_i, senior liabilities s_i and investment profit ρ_i of a bank i given assumptions $(i - iv)$ are all proportional to k, given respectively by

$$\lambda_i = \frac{f}{1-f}\,k, \quad (13)$$

$$s_i = \frac{f-\Lambda}{1-f}\,k, \quad (14)$$

$$\rho_i = \frac{(R_i-1)(1-\Lambda)}{1-f}\,k, \quad (15)$$

with $R_i = R$ if $i \neq i_0$ and $R_i = 0$ if $i = i_0$. The repayment equation (1) can therefore be written as

$$x_i = \left[\min\left\{\frac{(R_i-1)(1-\Lambda)+\Lambda}{1-f}\,k + \sum_{j\leftrightarrow i}\frac{x_j}{k}, rk\right\}\right]^+ \quad (16)$$

where the sum ranges over the neighbors j of i. Moreover, since in a Cayley tree all banks i at the same distance d from the shocked bank i_0 are equivalent, their repayments x_i must take a common value $x_{(d)}$, with $0 \leq x_{(1)} < x_{(2)} < \cdots \leq rk$. Thus (16) becomes

$$x_{(0)} = \left[\frac{-1+2\Lambda}{1-f}\,k + x_{(1)}\right]^+ \quad (17)$$

for the shocked bank itself and

$$x_{(d)} = \frac{(R-1)(1-\Lambda)+\Lambda}{1-f}\,k + \frac{x_{(d-1)}}{k} + (k-1)\frac{x_{(d+1)}}{k}, \qquad d \geq 1 \quad (18)$$

for its first, second and higher neighbors. From (17) and (18) it is easy to compute the first few critical degrees $k^*_{(d)}$.

By definition the first critical degree $k^*_{(1)}$ is such that

- only the shocked bank defaults, viz.

$$x_{(d)} = rk^*_{(1)} \quad \text{for} \quad d \geq 1, \quad (19)$$

- the first neighbors of i_0 are critical, viz.

$$x_{(1)} = \frac{(R-1)(1-\Lambda)+\Lambda}{1-f}\,k^*_{(1)} + \frac{x_{(0)}}{k^*_{(1)}} + (k^*_{(1)}-1)r = rk^*_{(1)} \quad (20)$$

Solving (17) and (20) for $k^*_{(1)}$ yields

$$k^*_{(1)} = \frac{r(1-f) - [r(1-f)+2\Lambda-1]^+}{(R-1)(1-\Lambda)+\Lambda}. \quad (21)$$

Similarly, the second critical degree $k^*_{(2)}$ corresponds to the situation where

- only the shocked bank and its first neighbors default, viz.

$$x_{(d)} = rk^*_{(2)} \quad \text{for} \quad d \geq 2, \quad (22)$$

- the second neighbors of i_0 are critical, viz.

$$x_{(2)} = \frac{(R-1)(1-\Lambda)+\Lambda}{1-f} k^*_{(2)} + \frac{x_{(1)}}{k^*_{(2)}} + (k^*_{(2)} - 1)r = rk^*_{(2)} \tag{23}$$

This gives (assuming $r < (1-2\Lambda)/(1-f)$, so that $x_{(0)} = 0$):

$$k^*_{(2)} = \frac{1}{2}\left(\sqrt{1 + \frac{4r(1-f)}{(R-1)(1-\Lambda)+\Lambda}} - 1\right). \tag{24}$$

Observe that $k^*_{(2)} = \mathcal{O}(k^{*1/2}_{(1)})$, hence the second critical degree $k^*_{(2)}$ grows much more slowly than the first critical degree $k^*_{(1)}$. This suggests that, except in the extreme case where $k^*_{(1)} \gg 1$ (which can happen only if $R \to 1$ and $f, \Lambda \to 0$), the direct propagation of failures to second and higher neighbors of the shocked bank is excluded in our model (within assumptions $(i-v)$).

Proof of Eq. (5)

Given the estimate (10), the expected number of failures among first neighbors of the shocked bank $\langle F_1 \rangle = \sum_{F_1 \geq 1} F_1 P_1(F_1)$ can be written as

$$\langle F_1 \rangle = \sum_{k \geq 1} p(k) \sum_{F_1=0}^{k} F_1 \binom{k}{F} q(k)^{F_1} [1-q(k)]^{k-F_1}. \tag{25}$$

Now, using Newton's binomial formula it is easy to show that, for any two numbers X and Y,

$$\sum_{F_1=0}^{k} F_1 \binom{k}{F_1} X^{F_1} Y^{k-F_1} = kX(X+Y)^{k-1}. \tag{26}$$

Using relation (26) with $X = q(k)$ and $Y = 1 - q(k)$ in Eq. (25) gives

$$\langle F_1 \rangle = \sum_{k \geq 1} kp(k)q(k). \tag{27}$$

Note that, for uncorrelated networks (for which $p(l|k) = lp(l)/z$), $q(k)$ is independent of k, hence $\langle F \rangle = zq$.

Proof of Eq. (12)

The Gauss hypergeometric function with parameters (a, b, c) is defined as the series

$$_2\mathcal{F}_1(a, b; c; \zeta) = \sum_{n=0}^{\infty} \frac{(a)_n (b)_n}{(c)_n} \frac{\zeta^n}{n!} \tag{28}$$

where $(m)_n = m(m+1)\dots(m+n-1)$ is the Pochhammer symbol and ζ is a complex number. (Note the potentially confusing notation: $_2\mathcal{F}_1$ is the Gauss hypergeometric function, and F is the number of failures induced by a shock.) Its asymptotic behavior in the limit of large c parameter is given by Watson's

expansion (see e.g. Olver et al., *NIST Handbook of Mathematical Functions*, Cambridge University Press 2010, p. 397), yielding in particular

$$\lim_{F\to\infty} {}_2\mathcal{F}_1(a,b;c+F;\zeta) = 1. \tag{29}$$

Using the connection formula

$${}_2\mathcal{F}_1(a,b;c;\zeta) = (1-\zeta)^{c-a-b}{}_2\mathcal{F}_1(c-a,c-b;c;\zeta), \tag{30}$$

this gives

$$\lim_{F\to\infty} {}_2\mathcal{F}_1(a+F,b+F;c+F;\zeta) = (1-\zeta)^{c-a-b}. \tag{31}$$

Consider the expression (10) for the distribution of failed first neighbors, assuming that F_1 is large enough (so that $q(k)$ has a constant value q), and plug in a power-law degree distribution of the form $p(k) \sim 1/(k)_\gamma$:

$$P_1(F_1) \quad \sim \quad q^F \sum_{k\geq F_1} \frac{1}{(k)_\gamma}\binom{k}{F_1}(1-q)^{k-F_1} = q^{F_1}\sum_{n\geq 0}\frac{1}{(F_1+n)_\gamma}\binom{F_1+n}{F_1}(1-q)^n. \tag{32}$$

Rewriting

$$\binom{F_1+n}{F_1} = \frac{(F_1+1)_n}{n!} \tag{33}$$

and

$$\frac{1}{(F_1+n)_\gamma} = \frac{1}{(F_1)_\gamma}\frac{(F_1)_n}{(F_1+\gamma)_n}, \tag{34}$$

we get

$$P_1(F_1) \sim \frac{q^{F_1}\,{}_2\mathcal{F}_1(F_1,1+F_1;\gamma+F_1;1-q)}{(F_1)_\gamma}. \tag{35}$$

Using the asymptotic formula (31), we arrive at

$$P_1(F_1) \sim \frac{q^{\gamma-1}}{F_1^\gamma}. \tag{36}$$

Mean number of failures: varying interest rate and leverage ratio

We studied the mean number of failures induced by a single shock within two network ensembles: Erdös-Rényi random networks and Barabási-Albert scalefree networks. Specifically, we compared our analytical estimate (5) with numerical results obtained by averaging over 10^4 networks for each value of the mean degree z.

Fig. S1 presents further results showing the effect of varying the leverage ratio Λ and the external interest rate R. While the agreement between theory (circles) and numerics (dots) remains qualitatively good for all considered values, we observe that systematic discrepancies—notably at low z—arise when Λ and R get large. This corresponds to regimes where the critical degree $k^* \gtrsim 15$. In such regimes, second and higher neighbors of the shocked bank are likely to fail, as the second critical degree $k^*_{(2)}$ becomes significantly larger than zero. It is an interesting challenge to extend our mean-field approximation so as to capture such higher-neighbor effects.

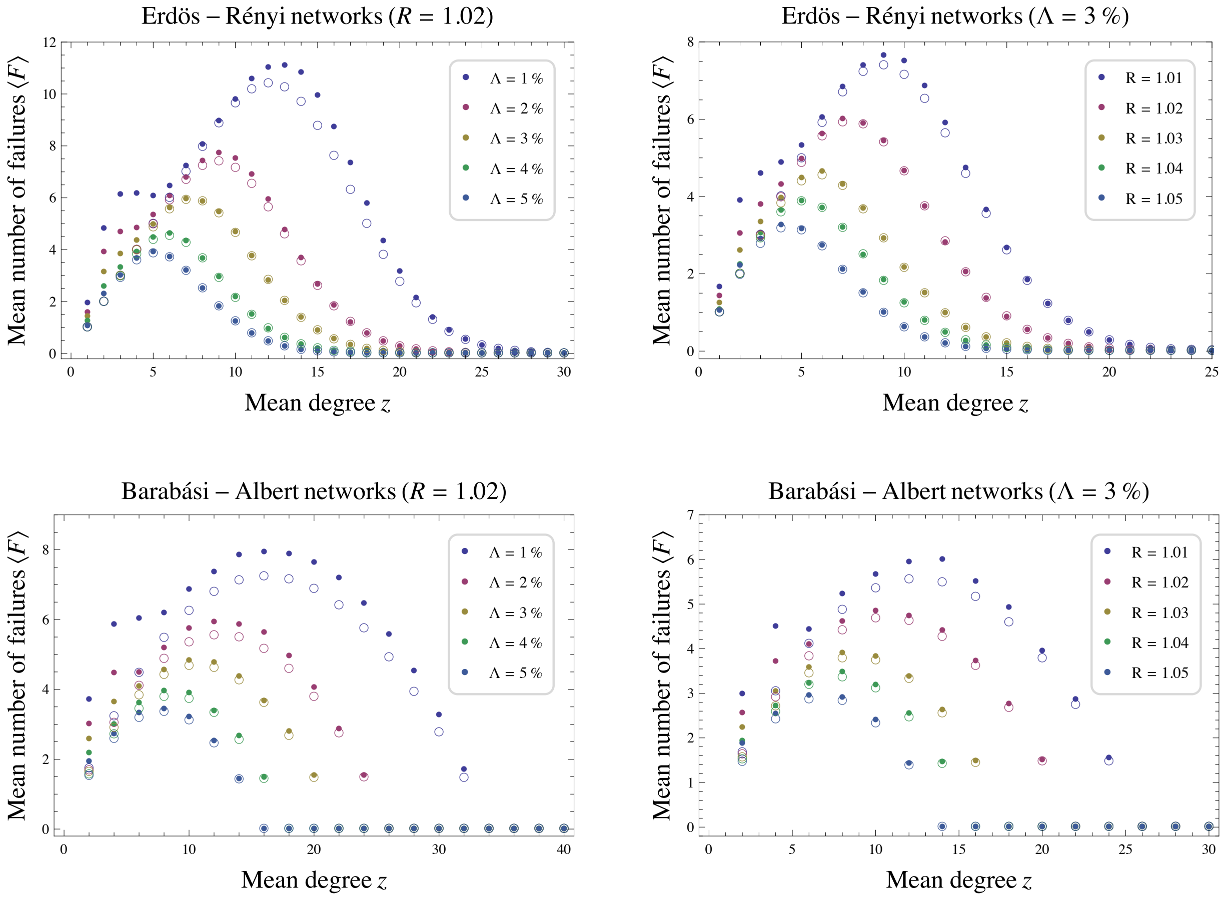

Figure B: Mean number of failures in ER (top) and BA (bottom) networks as a function of mean degree z, for $r = 1.01$ and $f = 50\%$. The circles represent our mean-field estimate (5), the dots represent the results of numerical averages over 10^4 networks with $N = 100$ banks. In the left column we vary Λ at fixed $R = 1.02$; in the right column we vary R at fixed $\Lambda = 3\%$ (right).
